# Supplementary material for: Zero-field nuclear magnetic resonance of chemically exchanging systems
Source: Nat Commun. 2019 Jul 5;10:3002. doi: 10.1038/s41467-019-10787-9 (PMC6611813; doi:10.1038/s41467-019-10787-9)
Supplement: Supplementary file 1 — Supplementary Information [file 41467_2019_10787_MOESM1_ESM.pdf]

Supporting Information

Zero-Field Nuclear Magnetic Resonance of Chemically Exchanging Systems

Danila A. Barskiy,*[,a,b] Michael Tayler,[c,&] Irene Marco,[d,x] John Kurhanewicz,[d] Daniel B. Vigneron,[d] Sevil Cikrikci,[a,e] Ayca Aydogdu,[a,e] Moritz Reh,[f] Andrey Pravdivtsev,[g] Jan-Bernd Hovener,[g] John Blanchard,[h] Teng Wu,[h] Dmitry Budker,[f,h] and Alexander Pines[a,b]

[a]Department of Chemistry, University of California - Berkeley, California 94720-3220, USA

[b]Materials Science Division, Lawrence Berkeley National Laboratory, Berkeley, California 94720-3220, USA

[c]University of Cambridge, Department of Chemical Engineering and Biotechnology, West Cambridge, CB3 0AS, UK

[&]Present address: The Institute of Photonic Sciences, Castelldefels 08860, Spain

[d]Department of Radiology and Biomedical Imaging, University of California – San Francisco, San Francisco, California 94158-2330, USA

[x]Present address: Institute for Bioengineering of Catalonia, The Barcelona Institute of Science and Technology, 08028 Barcelona, Spain

[e]Food Engineering Department, Middle East Technical University, 06800 Ankara, Turkey

[f]Department of Physics, University of California - Berkeley, Berkeley, California 94720-7300, USA

[g]Section for Biomedical Imaging, Molecular Imaging North Competence Center (MOIN CC), Department of Radiology and Neuroradiology, University Medical Center Schleswig-Holstein (UKSH), Kiel University, Am Botanischen Garten 14, 24118, Kiel, Germany

[h]Helmholtz Institute Mainz, Johannes Gutenberg-Universität, 55099 Mainz, Germany

Corresponding authors: barskiy@barkeley.edu (Danila A. Barskiy)

Table of Contents

Supplementary Figures 2
Supplementary Tables 8
Supplementary Notes 9

Supplementary Figures

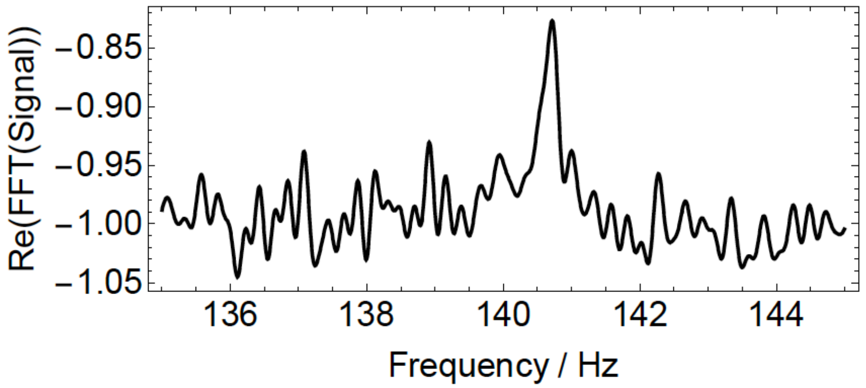

Supplementary Figure 1. ZULF NMR spectrum of neat [^{13}C]methanol (100 scans, prepolarization at 2 T) recorded using prototype portable ZULF NMR spectrometer. The same spectrometer with the same sensitivity was used for the detection of hyperpolarized [2-^{13}C]pyruvic acid.

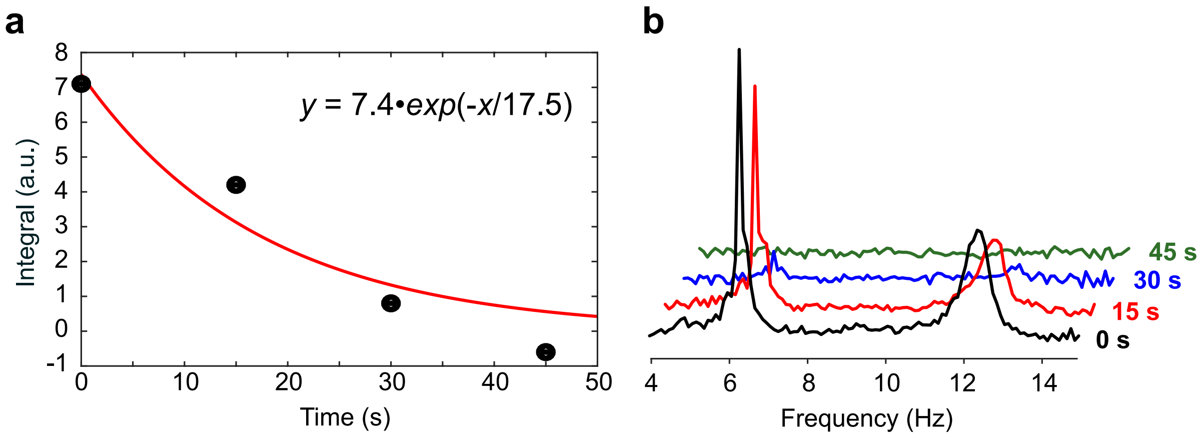

Supplementary Figure 2. a) Total integral of the ZULF NMR signal of [2-^{13}C]pyruvic acid as a function of time spent at Earth's magnetic field after hyperpolarization and b) corresponding ZULF NMR spectra.

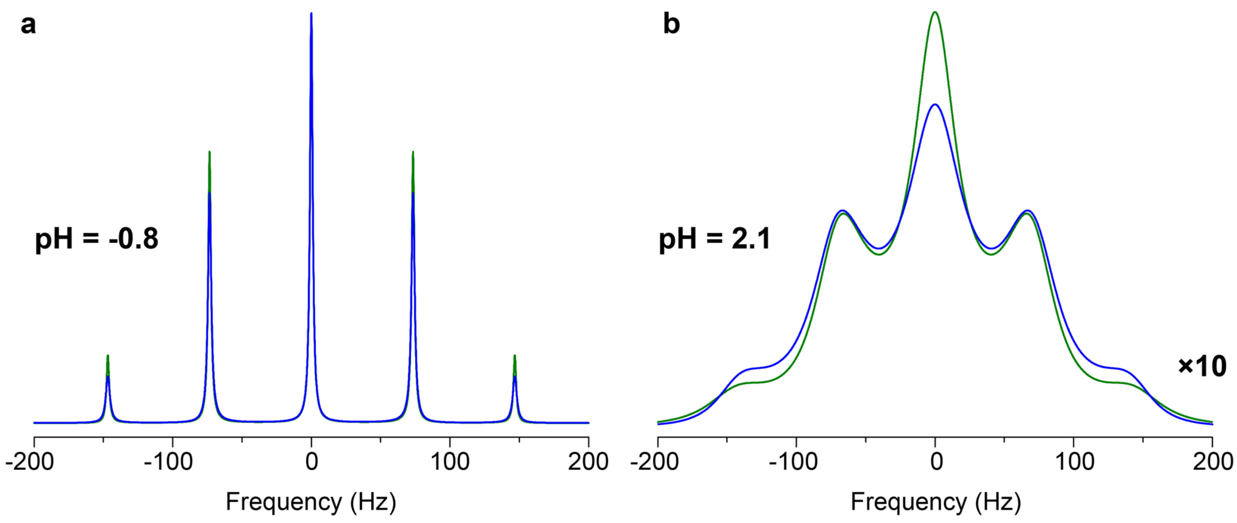

Supplementary Figure 3. Simulated ^{15}N NMR spectra of exchanging ^{15}N-ammonium ion for (a) pH = -0.8 and (b) pH = 2.1. Blue – simulation assuming simultaneous dissociation of all four hydrogen atoms, green – simulation assuming dissociation of a random hydrogen atom. Intrinsic NMR linewidth $FWHH_0 = 0.16$ Hz.

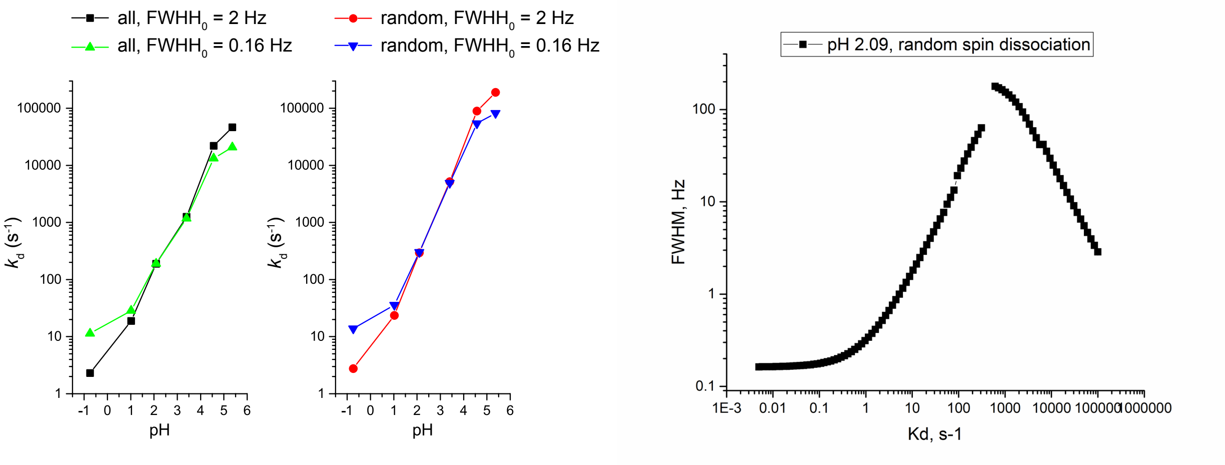

Supplementary Figure 4. Left – k_d values extracted from the experimental data using two simulation models described above ("all" vs. "random" proton dissociation) and different intrinsic linewidth values (2 Hz vs. 0.16 Hz). Right – full width at half height (FWHH) of a central NMR line as a function of proton dissociation rate (k_d) assuming random atom dissociation model. Discontinuities in the calculated values are due to changes in the number of Lorentzians used for fitting.

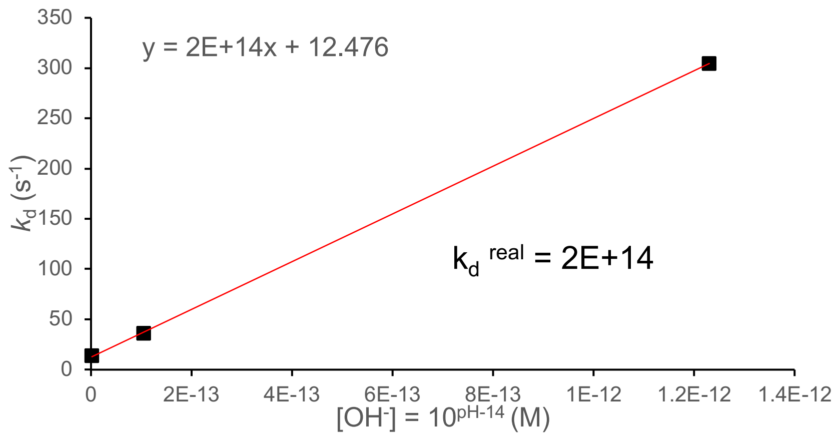

Supplementary Figure 5. Dissociation rate constant k_d plotted as a function of $[OH^-]$. One can see that for pH<3, k_d is linearly proportional to the concentration of $[OH^-]$ indicating bimolecular nature of the proton dissociation process.

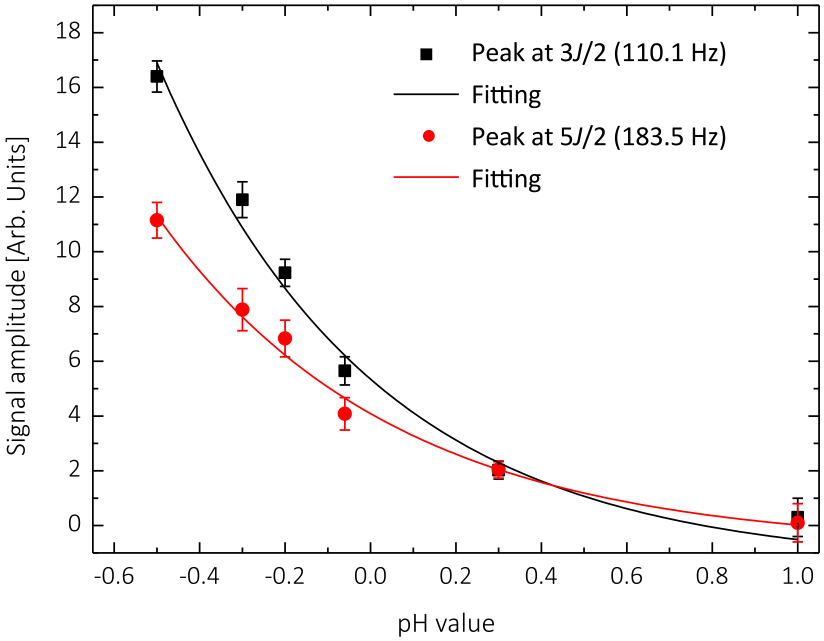

Supplementary Figure 6. Amplitude of the measured ZULF NMR peaks (black – peak at $3J_{NH}/2$, red – peak at $5J_{NH}/2$) as a function of pH in solution. Error bars are defined by fitting experimental signals with Lorentzian peaks.

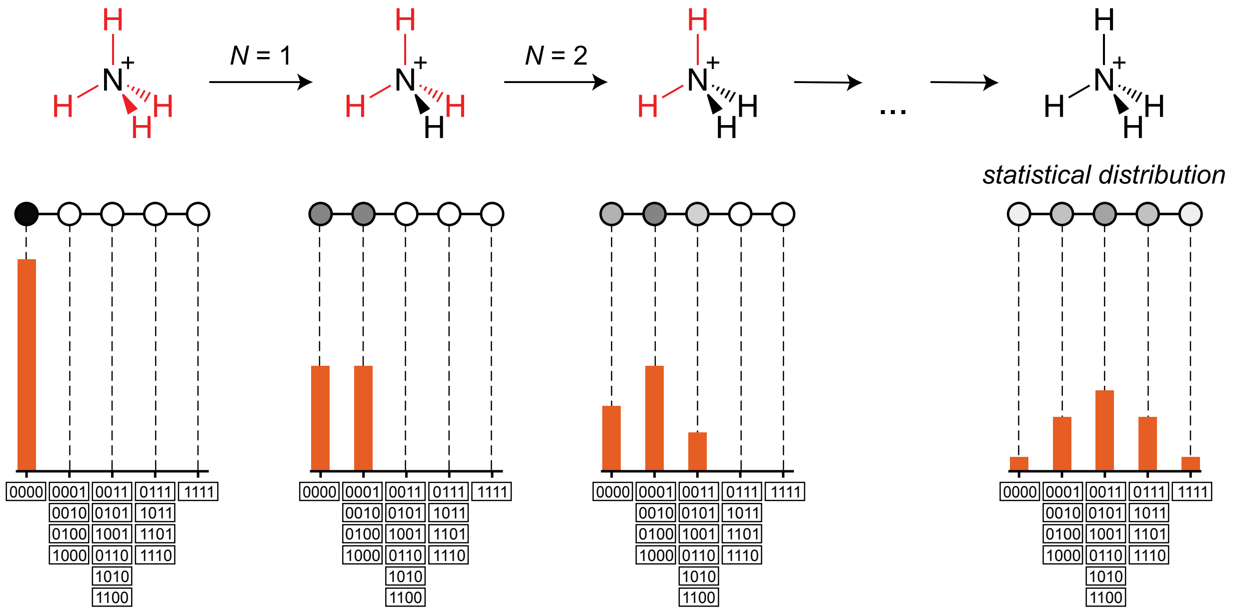

Supplementary Figure 7. Diagram of the chemical exchange process leading to the loss of nuclear "spin memory" in ammonia. Nuclear spin states are denoted in the computation basis, *i.e.*, $|\alpha\rangle = |0\rangle$, $|\beta\rangle = |1\rangle$. Initially, all four ^{1}H atoms are polarized (state $|\alpha\alpha\alpha\alpha\rangle$, or $|0000\rangle$). One exchange event returns hydrogen spin with equal probability of states $|0\rangle$ and $|1\rangle$.

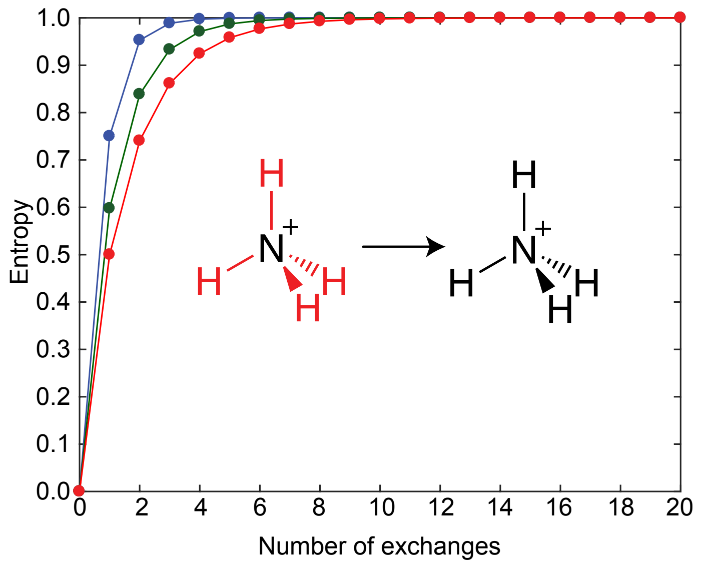

Supplementary Figure 8. Normalized entropy in the spin system as a function of the number of proton exchange events (in each exchange event, upcoming proton is considered to be unpolarized). Blue – exchange in hypothetical A_2X system, green – exchange in hypothetical A_3X system, Red – exchange in A_4X system.

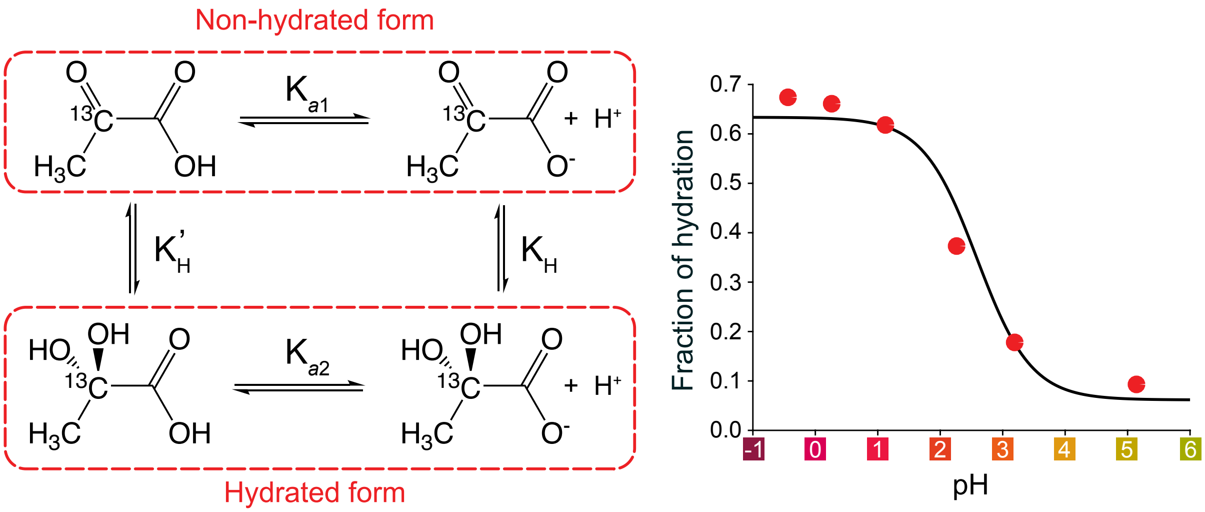

Supplementary Figure 9. Left - molecular diagram of the hydration and hydrogen exchange in pyruvic acid. Right – hydration fraction of pyruvic acid as a function of pH calculated from Supplementary Equation 18.

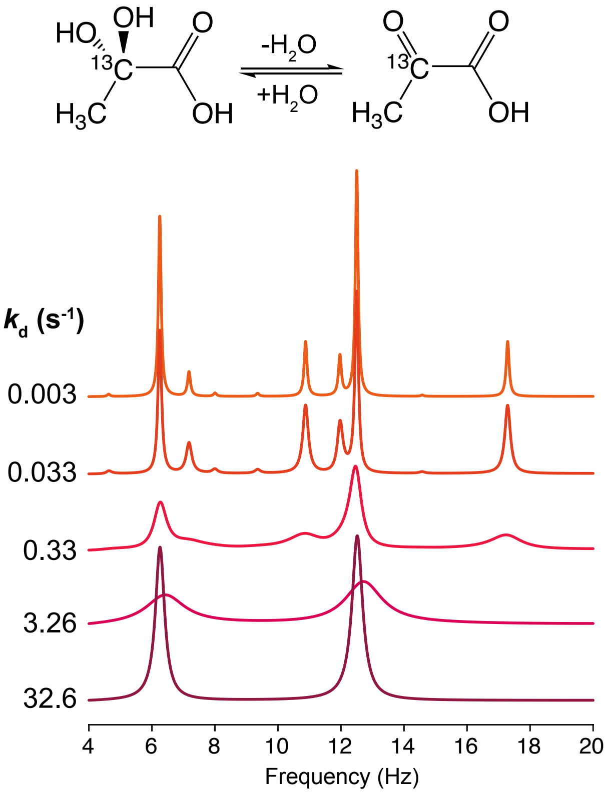

Supplementary Figure 10. Calculation of the effect of hydration on the line shape of zero-field NMR spectrum of [2-^{13}C]-pyruvic acid. The following exchange scheme was assumed: $(A_3X)B_2 \rightleftharpoons A_3X + 2B$, where $(A_3X)B_2$ is a spin system of 2,2-dihydroxypropionic acid, A_3X refers to a spin system of pyruvic acid and B refers to unpolarized ^{1}H atoms of water. Association exchange rates were calculated from the corresponding equilibrium constant $K'_H = k_a/k_d$.

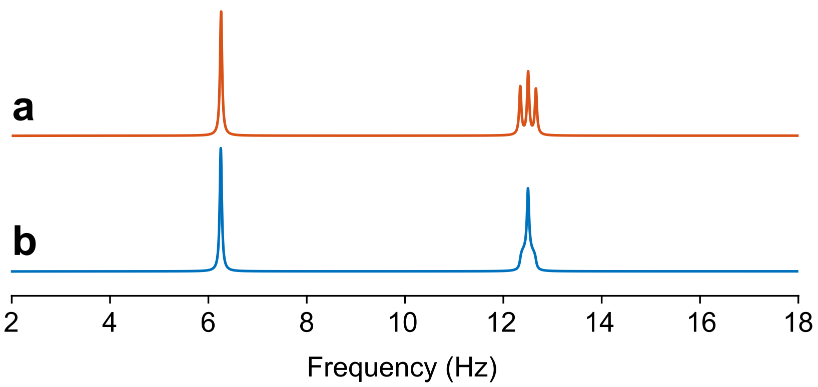

Supplementary Figure 11. Calculation of the effect of magnetic field inhomogeneity. a) Effect of the uniform magnetic field (10 nT) present along magnetometer sensitive axis; b) effect of the linear field (0-10 nT) gradient along magnetometer sensitive axis.

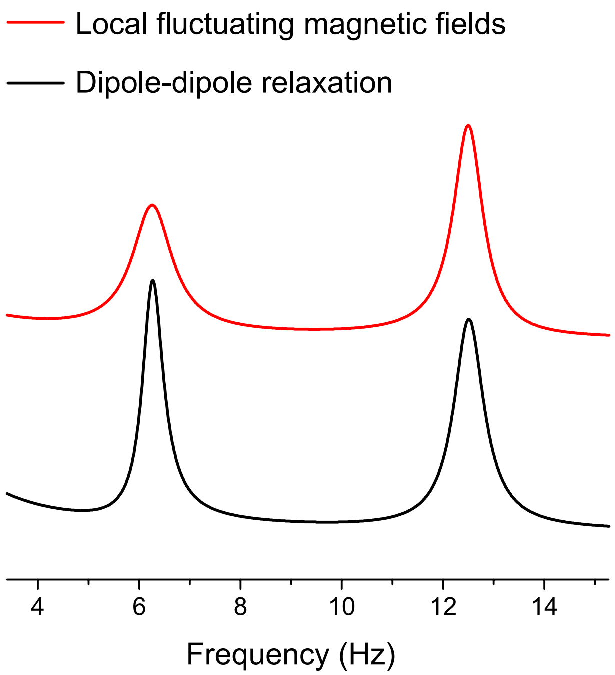

Supplementary Figure 12. Calculation of the effect of relaxation on the line shape of zero-field NMR spectrum of [2-^{13}C]-pyruvic acid. Top (red) – using the model of fluctuating magnetic fields (assuming high-field T_1 of 1 s)[2], bottom (black) – using the model of dipole-dipole (DD) relaxation. Parameters used for calculation using DD model – distance between ^{13}C and ^{1}H nuclei is 2.13 Å, distance between ^{1}H nuclei is 1.78 Å. Program for calculation is available online at [4].

Supplementary Tables

Supplementary Table 1. Integrals of ^{1}H NMR peaks corresponding to CH_3 groups of pyruvic acid ($\boldsymbol{S_1}$) and its hydrated form ($\boldsymbol{S_2}$) as a function of pH. Fraction of hydration is calculated as $\boldsymbol{S_2}/(\boldsymbol{S_1}+\boldsymbol{S_2})$.

**Measured pH**	**Integral (a.u.)**		**Hydration fraction**
	$\boldsymbol{S_1}$	$\boldsymbol{S_2}$	
-0.4	0.99	2.03	0.673
0.3	2.02	3.92	0.660
1.1	2.31	3.72	0.617
2.3	4.16	2.46	0.372
3.2	5.11	1.10	0.177
5.2	5.54	0.56	0.092

Supplementary Table 2. Rate constants k_d (s^{-1}) extracted from fitting simulated high-field ^{15}N NMR spectra to experimental data given the exchange model ("all" vs. "random" exchange) and different intrinsic linewidth values.

pH	All FWHM0=2 Hz	Random FWHM0=2 Hz	All FWHM0=0.1592Hz	Random FWHM0=0.1592Hz
-0.75	2.3	2.79	11.35	13.96
1.02	18.85	23.85	28.57	36.19
2.09	188.5	296.9	190.5	304.8
3.4	1256.6	5251.1	1179.3	4947.4
4.56	21991	89795.9	13275.9	54526
5.36	46495	190612.2	20689.7	82758.6

Supplementary Table 3. Linear interpolation for the rate constants k_d (s^{-1}) for pH ∈ [-0.5, 1.0] based on the fitting of the high-field NMR measurements.

pH	All FWHM0=2 Hz	Random FWHM0=2 Hz	All FWHM0=0.1592Hz	Random FWHM0=0.1592Hz
-0.5	4.6	2.8	11.3	13.96
-0.3	6.5	23.8	28.6	36.2
-0.2	7.4	297	190.5	304.8
-0.1	8.4	5250	1180	4947
0.3	12.1	89790	13280	54530
1.0	18.7	190610	20690	82760

Supplementary Notes

Values for the integrals of ^{1}H NMR peaks corresponding to CH_3 groups of pyruvic acid ($\boldsymbol{S}_1$) and its hydrated form ($\boldsymbol{S}_2$) as a function of pH are given in the **Supplementary Table 1**. Hydration fraction was calculated as $\boldsymbol{S}_2/(\boldsymbol{S}_1+\boldsymbol{S}_2)$ and plotted in **Figure 3b** of the main text.

Polarization of [2-^{13}C]pyruvic acid at the time of ZULF NMR detection was estimated by comparing the integrals of corresponding ZULF NMR peaks of the hyperpolarized sample with the integral of ZULF NMR peak of thermally polarized [^{13}C]methanol (**Supplementary Figure 1**):

$$P_{\mathrm{HP}} = \left(\frac{I_{\mathrm{HP}}}{I_{\mathrm{therm}}}\right)\left(\frac{N_{\mathrm{therm}}}{N_{\mathrm{HP}}}\right)\left(\frac{C_{\mathrm{therm}}}{C_{\mathrm{HP}}}\right)P_{\mathrm{therm}} = \left(\frac{700}{170}\right)\left(\frac{100}{1}\right)\left(\frac{24.0\ \mathrm{M}}{0.08\ \mathrm{M}}\right)\cdot 1.7\cdot 10^{-6} \approx 0.2, \quad (1)$$

where P_{HP} , P_{therm} , N_{HP} , N_{therm} , C_{HP} , C_{therm} are polarization, number of scans, and concentrations for hyperpolarized and thermal samples, respectively. Thermal polarization of neat [^{13}C]methanol was achieved at the magnetic field of 2 T. Given low SNR of the signal of thermally polarized sample, polarization percentage of ~20% is a rough estimate. However, this number correlates well with polarization percentage obtained by high-field NMR after ejection from the dDNP polarizer.

Let us consider an example of calculating NMR spectra of ^{15}N-ammonium as a function of pH. There are two ways of writing down the master equation (Equation 3 of the main text). One way is to assume simultaneous dissociation of all four hydrogen atoms:

$$\frac{d}{dt}\begin{pmatrix}\hat{\rho}_{\mathrm{X}}\\ \hat{\rho}_{\mathrm{A4X}}\end{pmatrix} = \begin{pmatrix}\hat{\hat{L}}_{\mathrm{X}} - W_a\hat{\hat{\mathbf{1}}}_{\mathrm{X}} & +k_d\hat{\hat{\boldsymbol{T}}}{}^{(\mathrm{A4X})}_{4\mathrm{A}}\\ +W_a\hat{\hat{\boldsymbol{D}}}{}^{(\mathrm{X})}_{4\mathrm{A}} & \hat{\hat{L}}_{\mathrm{A4X}} - k_d\hat{\hat{\mathbf{1}}}_{\mathrm{A4X}}\end{pmatrix}\begin{pmatrix}\hat{\rho}_{\mathrm{X}}\\ \hat{\rho}_{\mathrm{A4X}}\end{pmatrix}, \quad (2)$$

where $\hat{\rho}_{\mathrm{X}}$ and $\hat{\rho}_{\mathrm{A4X}}$ are density matrices corresponding to a nitrogen atom and ammonium ion, respectively. (A and X represent ^{1}H and ^{15}N nuclei, respectively). Liouvillian superoperators $\hat{\hat{L}}_{\mathrm{X}} = -i\hat{\hat{H}}_{\mathrm{X}} + \hat{\hat{R}}_{\mathrm{X}}$ and $\hat{\hat{L}}_{\mathrm{A4X}} = -i\hat{\hat{H}}_{\mathrm{A4X}} + \hat{\hat{R}}_{\mathrm{A4X}}$ describe coherent evolution (defined by Hamiltonian superoperators, $\hat{\hat{H}}_i\hat{\rho} = [\hat{H}_i, \hat{\rho}]$). For calculating Redfield relaxation superoperators, we used two relaxation models: (i) local fluctuating magnetic fields (step-by-step computational approach is given in Ref. 1) and (ii) intramolecular dipole-dipole interaction (the calculational approach is described in Refs. 2 and 3). The results of the calculations comparing the two relaxation models for [2-^{13}C]-pyruvic acid are shown in **Supplementary Figure 12**. Matrix $\hat{\hat{\boldsymbol{T}}}{}^{(\mathrm{A4X})}_{4\mathrm{A}}$ is a partial trace operator acting on the density matrix $\hat{\rho}_{\mathrm{A4X}}$ and resulting in the removal of subsystem 4A. The matrix $\hat{\hat{\boldsymbol{D}}}{}^{(\mathrm{X})}_{4\mathrm{A}}$ is a direct product superoperator representing formation of A_4X from X as a result of adding subsystem 4A to X.

The second (and more realistic) way of simulating proton dissociation considers the fact that each of the protons has equal probability for dissociation, therefore

$$\frac{d}{dt}\begin{pmatrix}\hat{\rho}_{\mathrm{A3X}}\\ \hat{\rho}_{\mathrm{A4X}}\end{pmatrix}=\begin{pmatrix}\hat{\hat{L}}_{\mathrm{A3X}}-W_a\hat{\hat{\mathbf{1}}}_{\mathrm{A3X}} & +k_d\hat{\hat{\boldsymbol{T}}}_{\mathrm{A}}^{(\mathrm{A4X})}\\ +W_a\hat{\hat{\boldsymbol{D}}}_{\mathrm{A}}^{(\mathrm{A3X})} & \hat{\hat{L}}_{\mathrm{A4X}}-k_d\hat{\hat{\mathbf{1}}}_{\mathrm{A4X}}\end{pmatrix}\begin{pmatrix}\hat{\rho}_{\mathrm{A3X}}\\ \hat{\rho}_{\mathrm{A4X}}\end{pmatrix}, \tag{3}$$

where we used a notation similar to that above. In this case, partial trace operator was taken as follows

$$\hat{\hat{\boldsymbol{T}}}_{\mathrm{A}}^{(\mathrm{A4X})}=\frac{1}{4}\left(\hat{\hat{\boldsymbol{T}}}_{\mathrm{A1}}^{(\mathrm{A4X})}+\hat{\hat{\boldsymbol{T}}}_{\mathrm{A2}}^{(\mathrm{A4X})}+\hat{\hat{\boldsymbol{T}}}_{\mathrm{A3}}^{(\mathrm{A4X})}+\hat{\hat{\boldsymbol{T}}}_{\mathrm{A4}}^{(\mathrm{A4X})}\right). \tag{4}$$

The explicit form of the matrices $\hat{\hat{\boldsymbol{T}}}_{\mathrm{A}i}^{(\mathrm{A4X})}$ can be found in Ref. 4. Interestingly, both simulation approaches produce similar result (see below).

High-field ^{15}N NMR spectra were calculated for the species A_nX by taking the Fourier transform of their time-dependent NMR signal $S(t)$, which was calculated as follows:

$$S(t)=\mathrm{Tr}\left\{\left(\hat{I}_x+i\hat{I}_y\right)\hat{\rho}_{\mathrm{AnX}}(t)\right\}. \tag{5}$$

Here $\hat{I}_x$ and $\hat{I}_y$ are nuclear spin operators corresponding to the spin X (written in the product operator basis of the spin system A_nX), and $\hat{\rho}_{\mathrm{AnX}}$ is a density matrix of the system A_nX.

Calculation of proton exchange in ammonium considers equilibrium constant determining the ratio of dissociation and association rates:

$$\mathrm{NH_4^+} \rightleftharpoons \mathrm{NH_3}+\mathrm{H^+}, \qquad \mathrm{K}_a=\frac{[\mathrm{NH_3}][\mathrm{H^+}]}{[\mathrm{NH_4^+}]}=\frac{k_d}{k_a}\,. \tag{6}$$

From Supplementary Equations 6, one finds the association rate W_a as

$$W_a=k_a[\mathrm{H^+}]=\frac{k_d}{\mathrm{K}_a}10^{\mathrm{pK}_a-\mathrm{pH}} \tag{7}$$

and the molar fractions of conjugate forms

$$x_{\mathrm{NH_4^+}}=\frac{1}{1+\mathrm{K}_a10^{-\mathrm{pH}}}, \qquad x_{\mathrm{NH_3}}=\frac{\mathrm{K}_a10^{-\mathrm{pH}}}{1+\mathrm{K}_a10^{-\mathrm{pH}}}\,. \tag{8}$$

Exchange rates were extracted from the simulation and fitting the experimental data (**Supplementary Table 2-3**). Both simulation approaches (simultaneous dissociation from ammonium of all four hydrogen atoms and dissociation of a random hydrogen atom) give similar results, i.e., an initial broadening of the resonances with increased k_d until they merge and subsequent narrowing of the single NMR resonance (**Supplementary Figure 3**).

Supplementary Figure 4 shows that two simulation models ("all" vs. "random" proton dissociation) do not differ significantly in the regime of slow exchange (pH < 3), and give similar values for k_d (**Supplementary Table 3**). In the fast exchange regime (pH > 4) the value of k_d is four times larger for the "random" dissociation model, as expected. Importantly, for the slow and fast exchange regimes, the linewidth is significantly affected by the intrinsic linewidth ($FWHH_0$). For the pH values below 3, dissociation is linearly proportional to the concentration of hydroxyl anions (**Supplementary Figure 5**) demonstrating the fact that the rate-determining step is transition of a hydrogen ion from ammonium ion to $[OH^-]$.[5]

Zero-field NMR spectra are calculated in a way similar to high-field (Supplementary Equation 5) but considering the effect of all spins in the system and considering different NMR detection geometry:

$$S(t) = \mathrm{Tr}\left(\left(\gamma_\mathrm{A} \sum_{i=1}^{n} \hat{I}_z^{\mathrm{A}_i} + \gamma_\mathrm{X} \hat{I}_z^{\mathrm{X}}\right)\hat{\rho}_{\mathrm{AnX}}(t)\right) ; \quad (9)$$

here $\hat{I}_z^{\mathrm{A}_i}$ and $\hat{I}_z^{\mathrm{X}}$ are nuclear spin operators corresponding to the spins A and X, respectively (written in the product operator basis of the spin system A_nX), and $\hat{\rho}_{\mathrm{AnX}}$ is the density matrix of the system A_nX.

Supplementary Figure 6 demonstrates the decline of the amplitude of the ^{15}N-ammonium as a function of the pH of the solution.

Since ZULF NMR signal of $^{15}NH_4$ is mainly determined by polarization of protons (due to much larger gyromagnetic ratio of ^1H compared to ^{15}N spins), one can determine the loss of proton polarization as a function of number of exchange events (**Supplementary Figure 7**). Let us compute how many proton exchange events are required for the system to go from 100% polarized state to the totally non-polarized state.

We start from composing a vector of state populations v_0 for the system with four identical spins:

$$v_0 = \begin{pmatrix} 1 \\ 0 \\ 0 \\ 0 \\ 0 \end{pmatrix} . \quad (10)$$

Here the states are $\alpha\alpha\alpha\alpha$, $\alpha\alpha\alpha\beta$, $\alpha\alpha\beta\beta$, $\alpha\beta\beta\beta$, $\beta\beta\beta\beta$ and spins are indistinguishable. Therefore, the thermal equilibrium spin state is

$$v^{\mathrm{eq}} = \begin{pmatrix} 1 \\ 4 \\ 6 \\ 4 \\ 1 \end{pmatrix} /16\ . \tag{11}$$

Let us introduce the "spin off" operator, $\mathrm{Pr}_{4\to3}$, that describes dissociation of a random spin in the system

$$\mathrm{Pr}_{4\to3} = \begin{pmatrix} 1 & 1/4 & 0 & 0 & 0 \\ 0 & 3/4 & 1/2 & 0 & 0 \\ 0 & 0 & 1/2 & 3/4 & 0 \\ 0 & 0 & 0 & 1/4 & 1 \end{pmatrix}. \tag{12}$$

The "spin on" operator describes association of the random unpolarized spin:

$$\mathrm{Pr}_{3\to4} = \begin{pmatrix} 1/2 & 0 & 0 & 0 \\ 1/2 & 1/2 & 0 & 0 \\ 0 & 1/2 & 1/2 & 0 \\ 0 & 0 & 1/2 & 1/2 \\ 0 & 0 & 0 & 1/2 \end{pmatrix}. \tag{13}$$

After N complete exchanges, the state v_N will be given by

$$v_N = (\mathrm{Pr}_{3\to4} \cdot \mathrm{Pr}_{4\to3})^N v_0\ . \tag{14}$$

In order to compare the final state with the thermal-equilibrium state, we define the normalized entropy (H) of the system as

$$H(v_N) = -\frac{1}{n}\sum_{k=1}^{2^n} p_k \log_2(p_k) = -\frac{1}{n}\sum_{i=1}^{5} v_i^N \log_2(v_i^N/Z_i)\,, \tag{15}$$

where v_i^N is the i-th element of the vector v_N, n is the number of spins (four, in the case of ammonium), Z_i is the partition function of the state corresponding to i. **Supplementary Figure 8** shows the entropy as a function of number of exchange events in a four-spin system (corresponding

to ammonium) as well as two hypothetical 3- and 2-spin systems. One can see that the entropy quickly grows as the number of exchange events increases.

One can see that 10 exchange events bring the system close to thermal equilibrium; this fact can be used to explain why the ZULF NMR signals of ammonium disappear under fast exchange rate (see text).

Proton dissociation from pyruvic acid (PA) results in formation of pyruvate (P) ion. The equilibrium constant for hydrogen dissociation for this process is known, $\mathrm{p}K_{a1} = 2.2$.[6] Hydrated form of pyruvic acid (PAH) is a weaker acid and upon proton dissociation forms pyruvate hydrate (PH) as characterized by the corresponding equilibrium constant, $\mathrm{K}_{a2} = 3.6$.[6] Equations describing equilibrium concentrations of the corresponding compounds are the following:

$$
\begin{aligned}
&\mathrm{PA} \rightleftharpoons \mathrm{P} + \mathrm{H}^+ \qquad && \mathrm{K}_{a1} = \frac{[\mathrm{P}][\mathrm{H}^+]}{[\mathrm{PA}]} = 10^{-\mathrm{pK}_{a1}} \,, \\
&\mathrm{PAH} \rightleftharpoons \mathrm{PH} + \mathrm{H}^+ && \mathrm{K}_{a2} = \frac{[\mathrm{PH}][\mathrm{H}^+]}{[\mathrm{PAH}]} = 10^{-\mathrm{pK}_{a2}} \,, \\
&\mathrm{P} \rightleftharpoons \mathrm{PH} && \mathrm{K_H} = \frac{[\mathrm{PH}]}{[\mathrm{P}]} = 0.064 \,, \\
&\mathrm{PA} \rightleftharpoons \mathrm{PAH} && \mathrm{K'_H} = \frac{[\mathrm{PAH}]}{[\mathrm{PA}]} = \alpha \mathrm{K_H} = 1.68 \,.
\end{aligned}
\tag{16}
$$

Here $\mathrm{K_H}$ is the equilibrium constant for the hydration of pyruvate and the parameter α describes the increase in hydration affinity for the pyruvic acid as compared to pyruvate. Indeed,

$$\frac{\mathrm{K}_{a1}}{\mathrm{K}_{a2}} = \frac{[\mathrm{P}][\mathrm{H}^+]}{[\mathrm{PA}]}\frac{[\mathrm{PAH}]}{[\mathrm{PH}][\mathrm{H}^+]} = \frac{\mathrm{K'_H}}{\mathrm{K_H}} = \alpha \,. \tag{17}$$

By substituting the numbers from the Supplementary Equation 16, one finds that $\alpha = 10^{\mathrm{pK}_{a2} - \mathrm{pK}_{a1}} \approx 26.3$.

One can easily derive from Supplementary Equation 16-17 that hydration fraction (χ) measured in high-field NMR is the following function of pH:

$$\chi = \frac{\mathrm{K_H}(\alpha + 10^{\mathrm{pH}-\mathrm{pK}_{a1}})}{\mathrm{K_H}(\alpha + 10^{\mathrm{pH}-\mathrm{pK}_{a1}}) + 10^{\mathrm{pH}-\mathrm{pK}_{a1}} + 1}. \tag{18}$$

As discussed in the main text, the fact that the NMR line at $2J_{\mathrm{CH}}$ is broader than the NMR line at J_{CH} have three possible explanations (see **Supplementary Figures 10-12**).

Supplementary References

1. Ivanov, K.; Yurkovskaya, A.; Vieth, H.-M., High resolution NMR study of T1 magnetic relaxation dispersion. I. Theoretical considerations of relaxation of scalar coupled spins at arbitrary magnetic field. *J. Chem. Phys.* **2008,** *129* (23), 234513.

2. Kowalewski, J.; Mäler, L., Nuclear Spin Relaxation in Liquids: Theory, Experiments, and Applications. In *Series in Chemical Physics*, Moore, H. J.; Spencer, N. D., Eds. CRC Press Taylor & Francis Group Boca Raton, FL. , 2006; Vol. 2, p 426

3. Grant, A. K.; Vinogradov, E., Long-lived states in solution NMR: Theoretical examples in three- and four-spin systems. *J. Magn. Reson.* **2008,** *193*, 177-190.

4. MOIN spin library, http://www.moincc.de/method-development/mr/moin-spin-library.

5. Emerson, M. T.; Grunwald, E.; Kaplan, M. L.; Kromhout, R. A., Proton Transfer Studies by Nuclear Magnetic Resonance. III. The Mean Life of the Amine-Water Hydrogen Bond in Aqueous Solution1a. *J. Am. Chem. Soc.* **1960,** *82* (24), 6307-6314.

6. Pocker, Y.; Meany, J. E.; Nist, B. J.; Zadorojny, C., Reversible hydration of pyruvic acid. I. Equilibrium studies. *The Journal of Physical Chemistry* **1969,** *73* (9), 2879-2882.
